# A re-engineered immunotoxin shows promising preclinical activity in ovarian cancer

**DOI:** 10.1038/s41598-017-17329-7

**Published:** 2017-12-22

**Authors:** Gwendlyn Kollmorgen, Klara Palme, Annette Seidl, Stefan Scheiblich, Fabian Birzele, Sabine Wilson, Christian Clemens, Edgar Voss, Martin Kaufmann, Klaus Hirzel, Natascha Rieder, Ben-Fillippo Krippendorff, Frank Herting, Gerhard Niederfellner

**Affiliations:** 1 Discovery Oncology, Pharmaceutical Research and Early Development (pRED), Roche Innovation Center, Munich, Germany; 2 Immunoassay Development Oncological Diseases, Roche Diagnostics GmbH, Munich, Germany; 3 Early Development & Rare Reagents, Roche Diagnostics GmbH, Munich, Germany; 4 Pathology and Tissue Analytics, Pharmaceutical Research and Early Development (pRED), Roche Innovation Center, Munich, Germany; 5 Pharmaceutical Sciences, Pharmaceutical Research and Early Development (pRED), Roche Innovation Center, Basel, Switzerland; 6 Bioinformatics, Pharmaceutical Research and Early Development (pRED), Roche Innovation Center, Munich, Germany; 7 Pharmaceutical Sciences, Pharmaceutical Research and Early Development (pRED), Roche Innovation Center, Munich, Germany

## Abstract

RG7787 is a re-engineered mesothelin-targeted immunotoxin with reduced immunogenicity composed of a humanized anti-mesothelin Fab fragment and a B-cell epitope silenced 24 kD fragment of *Pseudomonas* exotoxin A. High prevalence of mesothelin-positive cases and a large unmet medical need make ovarian cancer a promising indication for the clinical development of RG7787. However, ovarian cancer patients also frequently have elevated serum levels of the cancer antigen 125 (CA-125). In principle this could pose a problem, since the binding sites for CA-125 and RG7787 on mesothelin were reported to overlap. However, we show here that RG7787 can readily displace even excess amounts of CA-125 in different cellular assays. Moreover when tested *in-vitro* on a panel of 12 ovarian cancer cell lines, RG7787 had high cytotoxic activity on COV644, Caov-4, and SNU-119 cells and fully inhibited growth of EFO-21, KURAMOCHI, OVSAHO, and Caov-3 cells with potency values ranging from 1 to 86 pM. Finally, we evaluated the *in-vivo* efficacy of RG7787 in OvCa6668, a patient-derived ovarian cancer model with high levels of CA-125 expression. RG7787 had moderate monotherapy efficacy but in combination with standard chemotherapies (cisplatin, paclitaxel) achieved pronounced tumor regressions. In summary our data support clinical testing of RG7787 in ovarian cancer.

## Introduction

Immunotoxins represent surface antigen-targeted payload delivery approaches for tumor therapy^1^. They consist of an antibody fragment for tumor-selective targeting fused to a bacterial toxin, like *Pseudomonas* exotoxin A (PE), as effector moiety. Upon cellular uptake by receptor-mediated endocytosis the immunotoxin is intracellularly processed and the PE payload escapes to the cytosol, where it inhibits protein synthesis by ADP-ribosylation of eukaryotic elongation factor 2 (eEF2). This halts protein synthesis and causes cell death by apoptosis or necrosis. Their unique mode of action differentiates immunotoxins from currently developed antiproliferative antibody drug conjugates^2^, since blockage of protein synthesis, in contrast to inhibition of tubulin polymerization with maytansinoids or auristatins, also affects non-dividing tumor cells. All hallmarks of cancer depend on continuous resynthesis of protein components; therefore immunotoxins represent a highly potent, multilevel attack on tumors. So far, however, the clinical use of immunotoxins, particularly in solid tumor indications, has been hampered by their high immunogenicity. In the case of SS1P, the first mesothelin-targeted PE-based immunotoxin to enter the clinic, formation of neutralizing anti-drug antibodies (ADAs) was observed in 90% of patients after a single cycle of therapy^3^. To overcome this problem RG7787 consists of a humanized Fab fragment and a B-cell epitope silenced 24 kD minimal PE fragment^4^. Eliminating the PE domain II from the effector moiety also improved other properties relevant for clinical development as it decreased nonspecific toxicity and endowed resistance to degradation by lysosomal proteases^5^. A small clinical trial with chemo-refractory malignant mesothelioma patients has recently demonstrated that SS1P can achieve substantial clinical benefit when multiple cycles of treatment can be given^6^. In this trial, the ADA response directed against PE was attenuated by an immune preconditioning regimen. Pretreatment with a combination of the lymphocyte-depleting drugs pentostatin and cyclophosphamide allowed up to 6 treatment cycles. Some patients had major tumor responses that lasted for more than 20 months - well beyond the last treatment cycle.

Apart from mesothelioma, RG7787, is also a promising therapeutic agent for other solid tumor indications that highly express the tumor specific differentiation antigen mesothelin (MSLN), like ovarian and pancreatic cancer^7–9^. On normal tissue, mesothelin expression is restricted to differentiated mesothelial cells that line, as simple squamous epithelium, the main inner body cavities and organs (e.g. pleura, pericardium, and peritoneum). Because of this unique combination of high expression in different solid tumors and its complete absence from any vital normal tissues, mesothelin is being widely pursued for tumor-selective toxic payload delivery and cancer immunotherapy approaches^9,10^.

Pancreatic and ovarian cancer patients frequently also have high serum levels of the cancer antigen-125 (CA-125). Elevated levels of CA-125 can occur in most types of adenocarcinomas, particularly once they have established distant metastases. The highest serum levels of CA-125 are found in ovarian cancer patients, where levels sometimes reach >900 U/ml^11^. Around 80% of epithelial ovarian cancers show elevated CA-125 serum levels judged by a threshold criteria of >35 U/ml^12^ and the frequency of CA-125 positivity increases with high tumor stage (FIGO II, III, or IV)^13^. CA-125 binds to mesothelin and this interaction has been suggested to play a role for the ability of cancer cells to metastasize e.g. to the peritoneum^14–16^. The amino terminal region in mesothelin that is responsible for its interaction with CA-125 has been reported to overlap with the binding epitope of MORAb-009, a humanized version of the murine SS1 antibody^17–19^. MORAb-009 recognizes the same epitope as RG7787 and appears to be able to competitively disrupt the CA-125/mesothelin interaction as indicated by the fact that serum CA-125 levels increased markedly in all patients treated with MORAb-009 and rapidly decreased again after antibody therapy was stopped^20^. However, in the case of RG7787, therapy competition with excess CA-125 for binding to mesothelin could potentially pose a bigger problem, since (i) RG7787 only has a monovalent binding moiety and hence lacks the avidity effect that comes with the bivalency of an antibody; (ii) immunotoxins are dosed much lower than antibodies (c_max_ on the order of 1 µg/ml as compared to 200 µg/ml) (iii) the serum half-life of an immunotoxin is much shorter than that of an antibody (hours as compared to days). Therefore, we investigated whether abundance of CA-125 can negatively affect the ability of RG7787 to bind to and be taken up by mesothelin-positive tumor cells, thereby potentially preventing therapeutic success.

Surface plasmon resonance experiments confirmed that the binding sites for RG7787 and CA-125 on mesothelin overlap. However, in various biochemical and cellular assays we found that the interaction of CA-125 with mesothelin was not strong enough to block binding of the Fab moiety that targets RG7787 to tumor cells indicating that soluble CA-125 levels in patients cannot antagonize RG7787 treatment. In a small panel of ovarian cancer cell lines we did not observe an unusually high incidence of intrinsic PE resistance due to previously reported frequent loss of heterozygosity of DPH1. Seven out of 12 ovarian cancer cell lines were highly sensitive to the cytotoxic effects of RG7787 at picomolar concentrations. Finally we also performed an *in-vivo* efficacy study in OvCa6668, a patient-derived ovarian cancer model that expresses high levels of CA-125. In this model, combining RG7787 with standard chemotherapies led to tumor regressions and in particular combining RG7787 with paclitaxel resulted in highly synergistic efficacy. Taken together our data support clinical development of RG7787 in ovarian cancer.

## Results and Discussion

### Expression analysis of MSLN and CA-125

**Figure 1:**
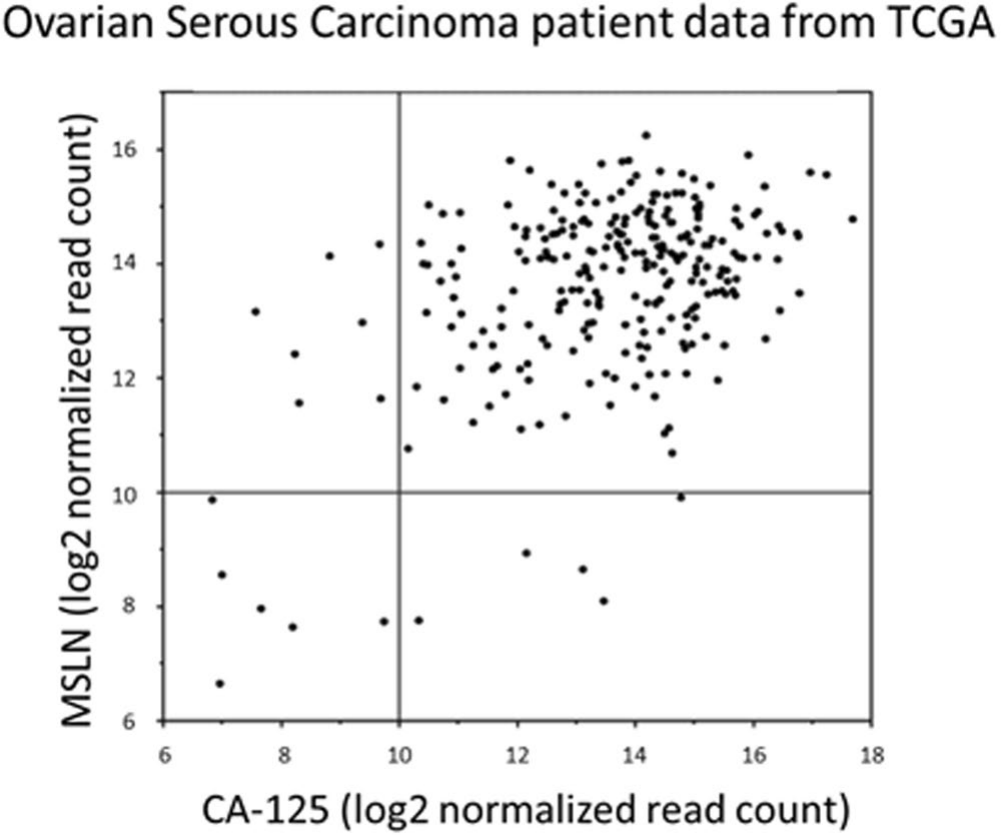
Expression of CA-125 and MSLN in ovarian cancer. The ovarian serous cancer cohort of The Cancer Genome Atlas (TCGA) was analysed for mesothelin (MSLN) and CA-125 expression. Log2 normalized read counts for CA-125 are plotted on the x-axis and for MSLN on the y-axis.

For a broad picture of how frequently high levels of CA-125 are co-expressed with mesothelin we mined the Cancer Genome Atlas (TCGA). Expression data for mesothelin and CA-125 were analyzed from ovarian serous carcinoma cases which represent the most common type of ovarian cancer with two thirds of all diagnosed patients. Figure 1 shows that 93% of these patients have high expression of both genes while only 5% show low expression of either mesothelin or CA-125 and merely 2% expressed both genes at low levels. We also analyzed the relative up-regulation of the mesothelin and CA-125 genes in various other solid tumor indications compared to corresponding normal tissues (Supplementary Figures S1a and S1b, respectively). Overall statistically significant overexpression of mesothelin was seen in stomach, colon, and rectum adenocarcinoma as well as bladder urothelial carcinoma, while for lung squamous cell carcinoma and two types of kidney cancer (chromophobe and renal clear cell carcinoma) mesothelin expression was reduced compared to normal tissue. For CA-125 tumor-specific overexpression reached statistical significance in breast invasive carcinoma, uterine corpus endometrioid carcinoma, and lung adenocarcinoma. The analysis demonstrates that high level co-expression of mesothelin and CA-125 is most prevalent in ovarian cancer, where the vast majority of patients express both genes at abnormally high levels, although it does not preclude that this phenomenon also occurs in certain subpopulations of the other solid tumor indications, e.g. 25% of peritoneal mesotheliomas have high CA-125 expression^21^. Based on this analysis RG7787 needs to be able to effectively compete with excessive amounts of CA-125 for binding to mesothelin on the surface of ovarian tumor cells in order to achieve a medical benefit.

### Surface plasmon resonance evaluation of RG7787 and CA-125 competition

**Figure 2:**
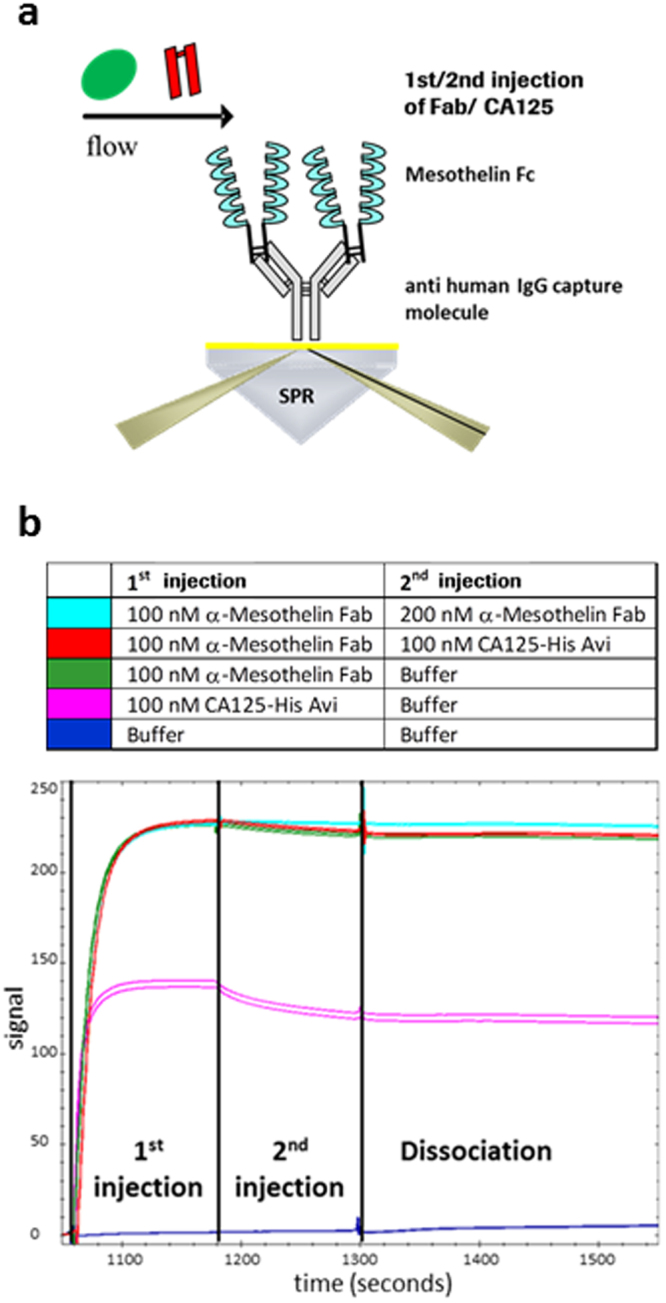
CA-125 competes with, but cannot displace anti-MSLN FAB from its epitope. **(a)** SPR assay schematic illustrating capture of the human MSLN extracellular domain as Fc fusion protein via an anti-huFc antibody coupled to the sensorchip. **(b)** No significant dissociation was observed (dark blue line, buffer injection). Both CA-125-HisAvi (purple line) and anti-MSLN Fab (green line) can bind to the captured MSLN-ECD but due to steric hindrance CA-125 reaches only partial saturation of binding sites, whereas the Fab reaches ~100% saturation as confirmed by no further signal increase upon a 2nd injection of a higher Fab concentration (light blue line). When CA-125 is offered in a second injection after saturation with the FAB (red line), no further binding occurs demonstrating that binding of both molecules to mesothelin is mutually exclusive.

We used surface plasmon resonance to confirm that CA-125 and RG7787 indeed have overlapping binding sites on mesothelin as indicated by Ma *et al*. 2012b and Kaneko *et al*. 2009. To this end a human mesothelin-Fc fusion protein was captured via an anti-human Fc antibody onto a Biacore sensor chip and then recombinant CA-125 or the Fab fragment present in RG7787 were added as analyte, respectively (Fig. 2a). Based on the calculated capture level of the human mesothelin-Fc fusion protein, CA-125 reached only partial saturation of binding sites, whereas the Fab fragment achieved complete saturation at the concentrations used. This is confirmed by the fact that a second injection of the Fab fragment at a higher concentration did not further increase the signal (light blue line). When, after saturation binding of the Fab fragment, CA-125 was offered in a second injection, the binding signal should have increased, if the binding sites for CA-125 and the Fab fragment do not overlap and both molecules can bind simultaneously to mesothelin. However, no increase in the binding signal was observed (red line versus green line in Fig. 2b). This confirms i) that the two molecules compete for sterically overlapping sites on mesothelin and ii) that CA-125 cannot displace pre-bound RG7787 Fab fragment from mesothelin, because displacement would have also resulted in a higher binding signal due to the substantially bigger protein mass of CA-125 versus the Fab fragment. In summary, binding of the targeting moiety of RG7787 and CA-125 to mesothelin is mutually exclusive and the Fab fragment of RG7787 binds with such high affinity that CA-125 cannot displace it in SPR competition experiments. We subsequently used two orthogonal methods to determine the absolute K_D_ value of RG7787 for mesothelin as “affinity in solution” (data not shown). By both methods the absolute affinity was found to be 12.5 pM. This extremely strong monovalent interaction between RG7787 and mesothelin is the result of an affinity maturation that has been performed to generate the SS1 clone from which the binding moiety of RG7787 is derived. The original clone SS had a >50 fold lower affinity^22,23^. When humanizing the SS1 Fab for use in RG7787 great care was taken to preserve its high binding affinity.

### RG7787 potency analysis in the presence of excess CA-125

**Figure 3:**
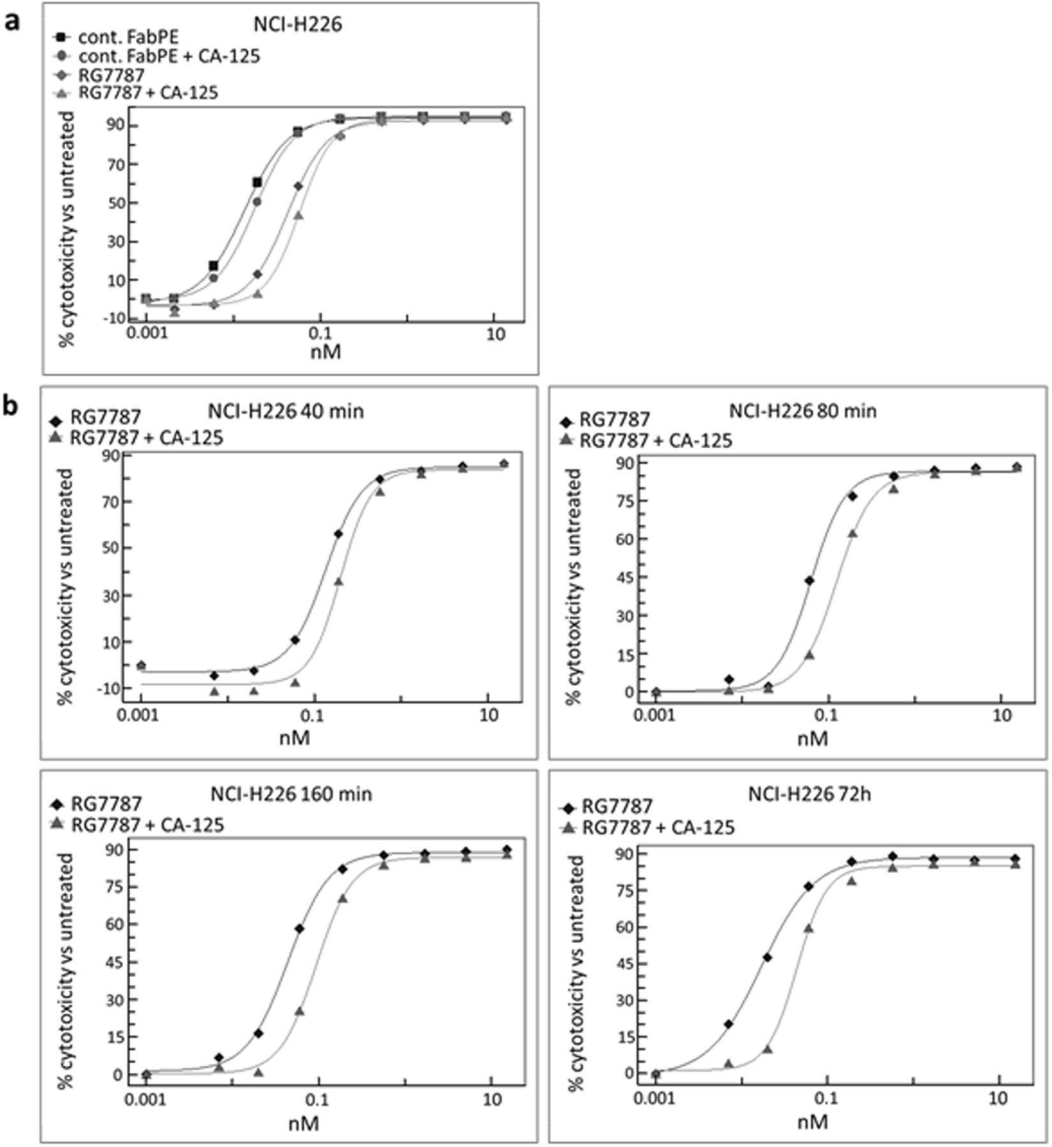
Potency of RG7787 in the presence of excess of CA-125. **(a)** Prior to the assay NCI-H226 cells were preincubated overnight with or without pleural fluid, which contained 29,500 U/ml of CA-125 as determined by Elecsys CA-125 measurement. Serial dilutions of RG7787 or a non-MSLN targeting control Fab-PE molecule with cytotoxic potency for NCI-H226 cells were added for 72 h. Cell viability was measured with CellTiter-Glo. Calculated IC50s with confidence intervals are as follows: RG7787 no CA-125: 0.042 nM (95% CI [0.0374, 0.0467]) versus RG7787 with CA-125: 0.058 nM (95% CI [0.0522, 0.0636]) and Fab-PE no CA-125: 0.013 nM (95% CI [0.0127, 0.0141]) versus Fab-PE with CA-125: 0.0176 nM (95% CI [0.0171, 0.0181]). **(b)** NCI-H226 cells were preincubated overnight with or without 28,000 U/ml of recombinant CA-125 as determined by Elecsys CA-125 measurement. The time that cells were exposed to RG7787 or a non-MSLN targeting control Fab-PE molecule was varied. For the 72 h time point the PE molecules were incubated with the cells for the duration of the experiment. For shorter exposure times the cells were incubated with the PE molecules for 40, 80, or 160 min and then the medium was removed and fresh medium was added for the remainder of the 72 h assay period. Cell viability was measured with CellTiter-Glo. The IC50s for RG7787 without and with CA-125, respectively, in ascending order of incubation length are: 40 min: 0.145 nM (95% CI [0.1185, 0.1522]) and 0.227 nM (95% CI [0.153, 0.2486]); 80 min: 0.064 nM (95% CI [0.0502, 0.0748]) and 0.127 nM (95% CI [0.1089, 0.1388]); 160 min: 0.044 nM (95% CI [0.0386, 0.0494]) and 0.095 nM (95% CI [0.0824, 0.1047]); and 72 h: 0.017 nM (95% CI [0.01482, 0.019]) and 0.044 nM (95% CI [0.0372, 0.0505]).

The effect of a large excess of CA-125 on the potency of RG7787 was analyzed in cell viability assays with the mesothelin-positive NCI-H226 cell line. This cell line, established from a pleural mesothelioma patient, was used as it represents a high level of endogenous tumor surface mesothelin expression (>100,000 molecules/cell)^24^, and it also allowed us to use another immunotoxin in parallel as control, which targets a different surface antigen of NCI-H226 cells. Prior to addition of the immunotoxins, NCI-H226 cells were preincubated overnight with or without pleural fluid containing 29,500 U/ml CA-125. Pretreatment with pleural fluid containing very high levels of CA-125 led to a minimal shift in the dose-response curves and slightly lower IC50 values of RG7787 treatment (Fig. 3a). Although these effects reached statistical significance based on the 95% confidence intervals (see legend for Fig. 3) they are too small to be biologically relevant. Moreover a similar slight, but statistically significant reduction in potency of the control immunotoxin that does not compete with CA-125 for binding to its target was observed (see “control Fab-PE” curves in Fig. 3a) indicating that the underlying cause is non-specific, and target-independent. Thus under the chosen experimental conditions we did not observe substantial target competition mediated impairment of the cytotoxic potency of RG7787 by even a large excess of CA-125.

In these first experiments the tumor cells were incubated with RG7787 for the entire 72 hours assay period. Therefore, a competitive effect of CA-125 for binding to MSLN might have been masked by continual low level unspecific uptake of RG7787. To exclude this possibility and to also mimic the relatively short circulation half-life of the SS1P immunotoxin (~30 min in mice, ~8 hours in man), the assays were repeated using shorter incubation times with the immunotoxin. In addition, we used recombinant CA-125 (28,000 U/ml) rather than pleural fluid for the pretreatment of target cells in a second set of experiments to exclude interference by other factors present in the pleural fluid. Binding of recombinant CA-125 to MSLN had been pre-verified by SPR in a pilot experiment (data not shown). RG7787 was added to the target cells only for either 40 min, or 80 min, or 160 min before the cell supernatant was replaced by fresh medium. Cell viability was read-out again at 72 hours. As expected, shorter incubation periods led to higher IC50 values as they leave less time for both target-mediated and unspecific uptake of the immunotoxin by cells. However, even under these more stringent experimental conditions the dose-response-curves of RG7787 with and without CA-125 pre-incubation differed at the most ~2 fold for all treatment durations suggesting that irrespective of its short plasma half-life RG7787 will be able to displace CA-125 from mesothelin in the clinical setting.

### Heterotypic cell adhesion assay in the presence of excess CA-125

**Figure 4:**
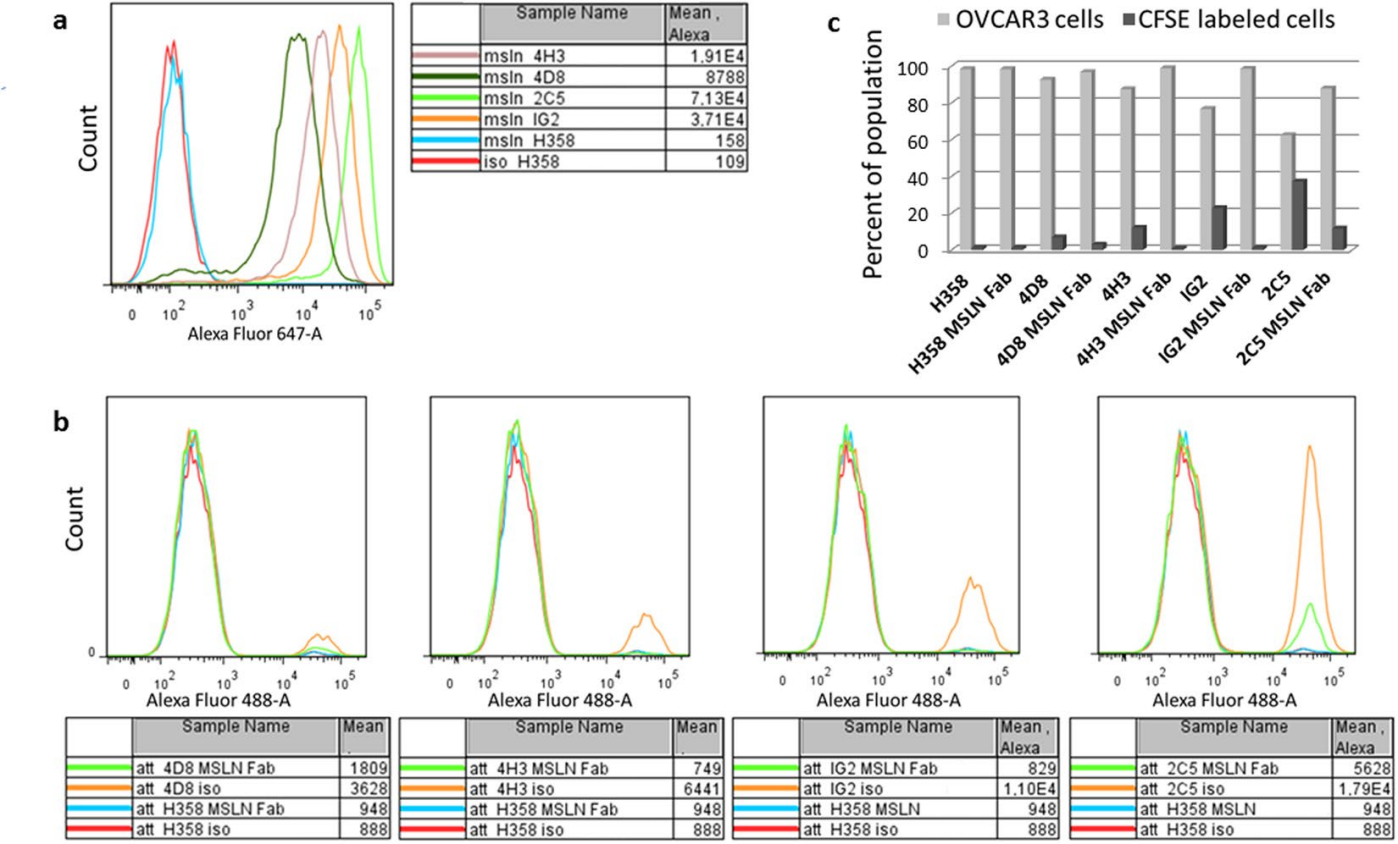
Heterotypic adhesion assay. (**a**) FACS quantification of mesothelin surface levels was done using humanized SS1P IgG and an anti-human Alexa Fluor 647 secondary antibody. While the FACS graph for parental H358 cells overlaps with that of the isotype control, increasing mesothelin surface staining was observed for the stably transfected clones 4D8 < 4H3 < 2C5 < IG2 **(b)** On day 1, 1 × 10^6^ OVCAR3 cells were seeded per well of a 12-well dish. 1 × 10^7^ of the parental and NCI-H358-MSLN clones were stained with 2.5 µM CFSE for 10 min at 37 °C and allowed to recover overnight. On day 2, the NCI-H358 cells were collected from the plates and 1 × 10^6^ cells were pre-incubated with either anti-MSLN Fab or a control Fab for 10 min. The NCI-H358 cells were then added to the OVCAR3 cells and interaction was accelerated by a brief centrifugation. After stringent washing, all of the remaining cells in the well were harvested and analyzed by FACS for CFSE positivity. **(c)** Bar graph depicting the relative percentages of CFSE labeled NCI-H358 cells vs unlabeled OVCAR3 cells.

Although soluble CA-125 was unable to compete with RG7787 for mesothelin binding in the above described *in-vitro* settings, the situation could be different for cell-cell interactions where both mesothelin and CA-125 are membrane-bound. A high density of both molecules on the surface of interacting cells could result in strong avidity effects. These could be further favored by the fact that each CA-125 molecule has multiple N-glycosylated regions that mediate mesothelin binding^15^. Such local avidity effects could impair the ability of RG7787 to effectively compete for its target. Therefore, we tested whether RG7787 can block the interaction between cells with high surface densities of CA-125 and mesothelin, respectively. A modified version of a heterotypic cell-cell adhesion assay previously described by Rump *et al*. 2004 was implemented using the same ovarian cancer cell line OVCAR3 that expresses high endogenous levels of CA-125, but combining it with a series of stably transfected NCI-H358 clones expressing different surface levels of mesothelin (Fig. 4a). This allowed us to use the parental NCI-H358 cell line lacking mesothelin expression as negative control for unspecific binding. In Fig. 4b the red line in all panels shows that parental NCI-H358 cells did not attach to OVCAR3 cells. With NCI-H358 clones of increasing cell surface mesothelin levels increasing levels of cell-cell attachment were observed: 4D8 < 4H3 < 1G2 < 2C5 (compare orange lines in the 4 panels of Fig. 4b) indicating that cell-cell interaction was mediated by CA-125 interaction with mesothelin. For the lower expressing clones 4D8, 4H3, and 1G2 adhesion to OVCAR3 cells was completely abrogated by pre-incubation with RG7787-Fab (Fig. 4b green lines). For the clone 2C5 that expresses extremely high levels of mesothelin (~5 million molecules/cell) only partial blockage could be achieved (Fig. 4b green line in right most panel). The relative percentages of OVCAR3 and NCI-H358 cells recovered in the attachment experiments were quantified by FACS and results are graphed in Fig. 4c. The proportion of NCI-H358 cells in the harvested cell population rose from <10% for the lowest mesothelin expressing clone 4D8 to >35% for the highest expressing clone 2C5. In the presence of competing RG7787 Fab no NCI-H358 cells were recovered by attachment to clones 4D8, 4H3, and 1G2, while for clone 2C5 the percentage was reduced from >35% to ~10%. In gastric cancer or triple negative breast cancer cell lines surface levels of mesothelin are typically in the range of 5,000 to 30,000 molecules per cell^25^, whereas in ovarian or pancreatic cancer cell lines they can exceed 50,000 molecules per cell^26^. The number of mesothelin molecules per cell on the NCI-H358 clones, whose attachment could be completely blocked, matches or even exceeds the highest range reported for endogenous mesothelin expression of cancer cell lines (140,000 for 4D8, 385,000 for 4H3, and 1,170,000 for 1G2). Thus RG7787 can effectively compete with the interaction between mesothelin on one cell type and CA-125 on another, even when both molecules are present on the cell surfaces at very high densities. This suggests such cell-cell interactions will not significantly impair tumor targeting by RG7787.

### Cytotoxic potency of RG7787 for ovarian cancer cell lines

Apart from competition with CA-125, there are other potentially cell type specific factors that can influence the sensitivity of ovarian cancer cells to PE-based immunotoxins like e.g. the rate of target internalization or the efficiency of intracellular trafficking and processing of a PE fusion protein. Moreover, in ~80% of early ovarian cancer cases allelic loss of a minimal region of 15 kb on chromosome 17p13.3 has been reported^27^. This allelic loss affects the tumor suppressor gene OVCA1^28^, which has recently been shown to encode the human Dph1 homologue. Dph1 together with Dph2 catalyzes the first step in diphthamide biosynthesis^29^, which is crucial for PE-sensitivity because the diphthamide-modified histidine 715 residue of the eukaryotic elongation factor 2 is the target of ADP-ribosylation by PE^1^. Therefore, if allelic loss of DPH1 were to result in incomplete diphthamide modification of eEF2, this could prevent complete inhibition of protein synthesis by PE and thus render such cells intrinsically non-responsive. To see, if an unusually high percentage of ovarian cancer cell lines are insensitive to RG7787 treatment despite being mesothelin positive, we tested in 10 point dose response titrations a panel of 12 ovarian cancer cell lines, preselected for mesothelin expression based on Affymetrix data. The results are listed in Table 1 as GI_50_ and percent maximum response values. Three cell lines (COV644, Caov-4, and SNU-119) have a maximum effect of ~200% GI indicating that no cells survived RG7787 treatment. With low single digit picomolar GI_50_ values COV644 and Caov-4 were among the most sensitive of a wider test panel of 40 cancer cell lines from different solid tumor types. Five other ovarian cancer cell lines were fully growth inhibited by RG7787 with potencies ranging from 9 pM to 0.3 nM. Only for three ovarian cancer cell lines (59M, OV56, COV504) a GI_50_ was not reached within the dose range used (up to 50 nM). This response profile is comparable to that of similar panels of 13 lung cancer cell lines and 11 pancreatic cell lines that we have tested (results not shown). In agreement with this finding that PE insensitivity is not observed more frequently for ovarian cancer cell lines than for other solid tumor cell lines, gene expression analysis did not show significant differences in DPH1 transcript levels between the different ovarian cancer cell lines (also listed in Table 1). Information on gene copy number variation available for the cell lines OVCAR3 and EFO-21 suggests that OVCAR3 indeed has loss-of-homozygosity for DPH1 and EFO-21 for DPH3 and DPH4. The example of OVCAR3 cells suggests that loss of a single allele of DPH1 is compensated by upregulation of transcription from the remaining allele and hence does not lead to intrinsic PE resistance. In line with this we recently could show that inactivation of one DPH1 allele in MCF7 cells by a gene-specific zinc-finger nuclease, was not sufficient to confer immunotoxin resistance, despite leading to a detectable amount (~15%) of non-diphthamide modified eEF2 molecules^30^. In summary the *in-vitro* potency results with a panel of cell lines suggest that ovarian cancer can be a promising clinical indication for RG7787 therapy.

**Table 1:** Potency of RG7787 in ovarian cancer cell lines.

Cell line	EC50 (nM)	GI50 (nM)	Max response (GI %)	DPH1 (Log2 Expression)
COV644	0.002	0.001	199	3.27
SNU-119	0.014	0.009	196	Not available
Caov-4	0.003	0.002	183	5.025
KURAMOCHI	0.009	0.009	108	5.82
Caov-3	0.086	0.11	90	Not available
EFO-21	0.03	0.046	90	4.26
OVSAHO	0.034	0.057	81	4.04
JHOS-2	0.297	0.67	75	Not available
OVCAR-3	1.1	8	59	3.56
59M	41	NA	31	3.59
COV504	0.614	NA	29	3.51
OV56	0.031	NA	23	3.47

The half maximal effective concentration (EC_50_) of RG7787 is listed for each cell line. The percent growth inhibition (GI %) is shown on the right. 100% GI represents complete cell growth inhibition. Values above 100% indicate that due to cell death there are fewer cells present at the end of the assay than at the start. When available the log2 expression value for the DPH1 gene was also included for each cell line.

### *In vivo* efficacy of RG7787 in an ovarian cancer PDX model

**Figure 5:**
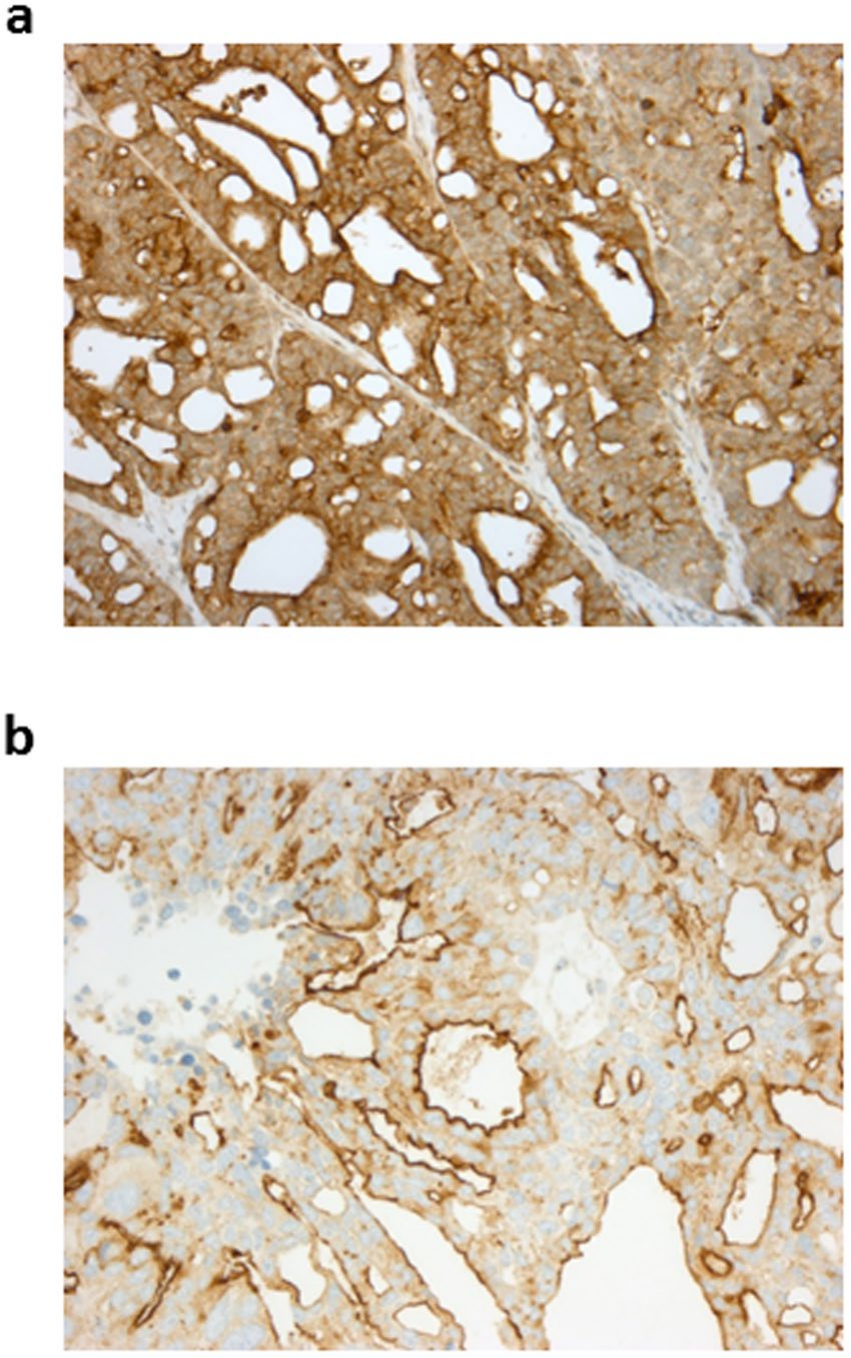
IHC staining of OvCa6668 tumors. Formalin fixed OvCa6668 tumors were stained for MSLN **(a)** or CA-125 **(b)**. For both proteins predominantly an apical staining pattern is observed. For MSLN 80% of tumor cells are positive (staining intensity +++). For CA-125 apical membranes stain positive (staining intensity ++−+++) also in 80% of tumor cells, while in 30% weaker cytoplasmic staining is observed.

Our *in-vitro* results suggested that RG7787 could offer a new therapeutic option for ovarian tumors, although most co-overexpress mesothelin and CA-125. Hence, we tested its efficacy in OvCa6668, a patient derived ovarian cancer xenograft model, in which we confirmed by immunohistochemistry high levels of both molecules. A strong apical membrane staining pattern was observed for MSLN in 80% of OvCa6668 tumor cells (Fig. 5a) and the same tumor samples also showed in 80% of the tumor cells moderate to strong apical membrane staining for CA-125 and cytoplasmic CA-125 positivity in 30% of the tumor cells (Fig. 5b).

Taxane and platinum based chemotherapy combination regimen are the standard of care treatments for ovarian cancer; therefore, we tested the efficacy of RG7787 not only as monotherapy but also in combination with paclitaxel and cisplatin. Both, paclitaxel (given at 6 mg/kg on 5 consecutive days) and cisplatin (given at 2 mg/kg once per week for two weeks) were well tolerated and did not cause any significant body weight loss. However, two cycles of RG7787 monotherapy (given at 2 mg/kg every other day three times per week for two weeks) caused up to 10% body weight loss. Dual combinations of RG7787 with either cisplatin or paclitaxel were borderline tolerable causing maximum body weight loss of up to 18% and resulting in death of two animals in the latter treatment group.

**Figure 6:**
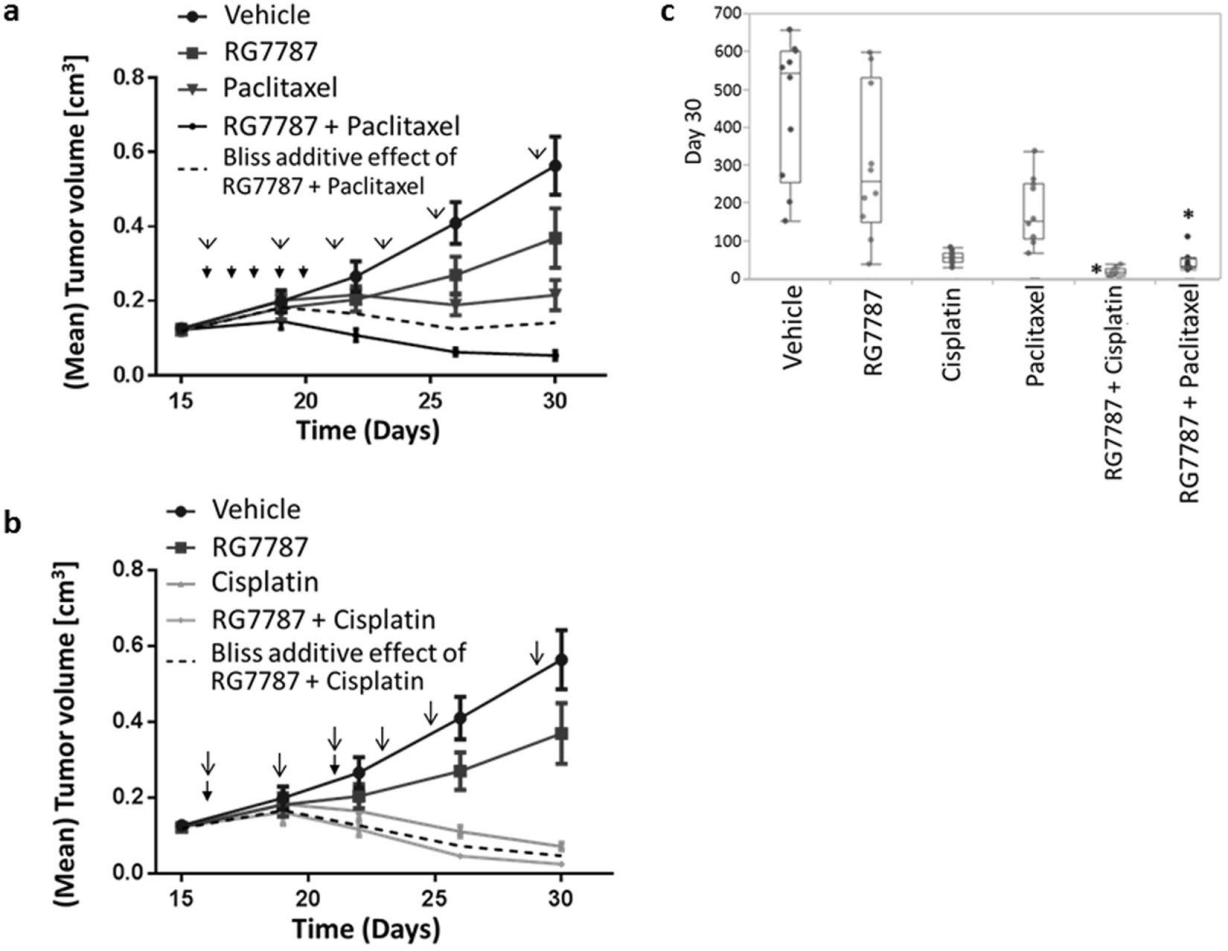
Efficacy of RG7787 as monotherapy and in combination with standard chemotherapies in the OVCa6668 patient-derived ovarian cancer model. OvCa6668 xenograft tumors were inoculated in NMRI nu/nu female mice. One day after randomization, treatment was started with either paclitaxel (6 mg/kg iv; q1dx5) or RG7787 (2 mg/kg iv; q2dx3 2 days off, q2dx3 2 days off, plus 7th treatment) or cisplatin (2 mg/kg ip; q7dx2) or combinations of various agents. The mean tumor volumes were plotted over time. Treatment time points are indicated by arrows and triangles. (**a**) Tumor growth curves are shown for combination therapy with RG7787 plus paclitaxel and the respective monotherapies and vehicle control group. From day 18 onwards the mean tumor volumes of the combination therapy group were substantially reduced as compared to the effect calculated using the bliss additivity model (dashed line). (**b**) Tumor growth curves are shown for combination therapy with RG7787 plus cisplatin and the respective monotherapies and vehicle control group. The mean tumor volumes of the combination therapy group begin to slightly separate from the calculated tumor growth curve based on the bliss additivity model (dashed line) only after day 23. (**c**) Box and whisker plots for the tumor volumes observed for the different treatment groups on day 30 (end of study date). For both combination therapy groups, asterisks mark where the mean tumor end volume based on the bliss additivity calculation lies relative to the 95% confidence interval of the actual measurements (represented by the box).

As single agent RG7787 caused a 40% reduction in median tumor volume versus the vehicle control group at day 30 when the study was terminated, however due to high variability within both groups this difference did not reach statistical significance (see boxplot in Fig. 6c). In monotherapy both paclitaxel and cisplatin were more efficacious achieving median tumor volume reductions of 69% and 87%, respectively (p values versus vehicle group 0.0337 and 0.0025, respectively). However, the combination of RG7787 and paclitaxel was not only statistically significantly better than each agent alone (p 0.0199 in both cases), but even had a more than additive effect on tumor growth inhibition (see calculated bliss additivity curve in Fig. 6a). This combination achieved tumor regressions, while tumors continued to grow, albeit at a reduced growth rate, when treated with either agent alone. As shown in panel c of Fig. 6 the expected tumor volume at day 30 for a purely additive effect lies far outside the 95% confidence interval for the measured values of the combination group. Combining cisplatin plus RG7787 also outperformed monotherapy with either RG7787 (p 0.0025) or cisplatin (p 0.0034), however the effect of this combination barely exceeded mere additivity (see calculated bliss additivity curve in Fig. 6b). This is also indicated in Fig. 6c where the asterisk marking the expected purely additive effect for this combination lies very close to the upper limit of the 95% confidence interval of the experimentally determined volumes. A second study using a lower, suboptimal dose of cisplatin (1 mg/kg rather than 2 mg/kg) confirmed that the RG7787/cisplatin combination has only an additive effect on tumor growth (data not shown).

Based on these data combining RG7787 with standard of care chemotherapies, but in particular with taxane treatment, appears to offer a promising new treatment option for ovarian cancer patients. Also for other solid tumor types synergistic effects of combined treatment with an immunotoxin and a taxane have been reported^31–33^. Several different mechanisms of interaction between these two types of agents have been demonstrated: (i) Taxol treatment can reduce the extent of shedding of mesothelin^34^ (the same might not apply for other surface targets of immunotoxins^35^). (ii) Tumor uptake *in-vivo* is usually a rate-limiting step for any immunotoxin therapy due to the short circulation half-life of immunotoxins and can be enhanced by taxol treatment^36^. The underlying mechanism is unclear although based on the observation that the effect requires tumor cells to be taxol-sensitive, it has been proposed that reduced interstitial pressure in the tumor upon elimination of tumor cells by taxol plays a role. However, the fact that we observed only additivity for combining RG7787 with cisplatin, but synergistic effects with RG7787 plus paclitaxel, although both agents had good monotherapy efficacy, suggests that this cannot be the only explanation. iii) it was recently found that taxol synergizes with RG7787 also *in-vitro*, but only in certain cancer cell lines^31^. Intriguingly, taxol does not enhance protein synthesis inhibition by RG7787, nor does it synergize with cycloheximide, an unrelated protein synthesis inhibitor. It appears that in certain tumor cells taxol lowers the threshold for apoptosis induction by the immunotoxin. Understanding the molecular basis of this sensitization would be extremely valuable for developing clinical response prediction biomarkers that could identify the patient population that profits most from this promising novel treatment option for ovarian cancer.

## Materials and Methods

### Cell lines and reagents

NCI-H226, NCI-H358 and NIH:OVCAR3 cells were obtained from ATCC. The ovarian cancer cell lines screened for RG7787 sensitivity at Horizon Inc. were obtained from the following providers: ATCC (Caov-3, Caov-4, and OVCAR3), Public Health England’s European Collection of Authenticated Cell Cultures (59M, COV504, and OV56), Health Science Research Resources Bank (KURAMOCHI and OVSAHO), Sigma (COV664), DSMZ (EFO-21), Riken (JHOS-2) and Korean Cell Line Bank (SNU-119).

Pleural fluid containing CA-125 at 183,600 U/ml was purchased from ADVY Chemical (Mumbai, India). CellTiter-Glo® reagent was from Promega (Mannheim, Germany). 293Fectin, cell culture medium, 6-carboxy-fluorescein succinimidyl ester (CFSE), and pcDNA 3.1 vector were obtained from Thermo Fisher Scientific (Darmstadt, Germany). For surface plasmon resonance (SPR) measurements, CM5 Sensor Chips, Amine Coupling Kit, Human Antibody Capture Kit, and 10x HBS-N-buffer from GE Healthcare were used (Dornstadt, Germany). PBS 10x, Tween 20, and BSA were from Roche (Sigma-Aldrich distributor; Taufkirchen, Germany). RG7787 has been previously described^26^ and was produced at Roche from inclusion bodies by refolding^37^.

If not otherwise indicated, all experiments were repeated at least twice.

### Preparation of CA-125 pleural fluid

After thawing the CA-125 containing pleural fluid, it was dialyzed (MWCO 25,000) against RPMI 1640 (3 × 1 l) and in the last step against 100 ml of RPMI + 10% FCS. Then it was sterilized by passage through a Millipore 0.22 µm PVDF hydrophilic syringe filter, aliquoted, and frozen at −80 °C.

### CA-125 cloning and expression

First cDNA was prepared from mRNA isolated from OVCAR3 cells. A portion of the CA-125 gene corresponding to the extracellular domain (amino acids 21051-22152), was amplified from that cDNA with the following primer pair: 5′-tctagagctagccaccatgctggacagagagcggctg-3′ and 5′-tctagagcggccgcgggaaggtcagaattccca-3′. The amplified gene was cloned into the Nhe1 and Not1 sites of a variant of the pcDNA 3.1 vector that had been modified to C-terminally add His- and Avi-tags. After obtaining correct clones, the sequence encoding the signal peptide from CD54 (atggctccca gcagcccccg gcccgcgctg cccgcactcc tggtcctgct cggggctctg ttcccaggac ct ggcaatgcc) and a portion of the CA-125 gene were inserted at the 5′ end using the Nhe1 vector site and an internal Xho1 site of the CA-125 sequence.

In order to biotinylate CA-125 during expression, the above vector (90%) was co-transfected with a plasmid containing the BirA (Bifunctional ligase/repressor BirA) gene (10%) into HEK-293F cells (600 ml culture with 2 × 10^6^ cells/ml) using 293Fectin according to the manufacturer’s manual (Thermo Fisher Scientific, Darmstadt, Germany). After 3 h, biotin dissolved in 100 mM Na_2_HPO_4_ was added to a final concentration of 50 µM. The cells were incubated for 7 days then the supernatant was collected and filtered before purification on a His-trap column.

### Surface plasmon resonance (SPR) measurement

All experiments were performed on Biacore T200 instruments and evaluated with the corresponding software 2.0 from GE Healthcare. The running buffer applied was phosphate-buffered saline with 0.05% Tween 20. Running buffer supplemented with 1 mg/ml BSA was used for dilution. Standard amine coupling was performed as recommended by GE Healthcare. Coupling was done in HBS-N (10 mM HEPES, 150 mM NaCl, pH 7.4) buffer. Activation was performed by a 4:1 molar ratio of EDC/NHS. Ligand was diluted in coupling buffer containing 10 mM sodium acetate, pH 5.0. Finally, residual activated carboxyl groups were blocked by injection of 1 M ethanolamine (pH 8.5). Binding signals were double referenced against blank buffer and a flow cell containing no ligand. An Fc-fusion protein of human mesothelin was captured via anti-human IgG (Fc) antibody (GE) that was amine coupled on a CM5-Chip surface. Anti-mesothelin Fab and CA-125-His-Avi were injected consecutively and conversely using two dual injects with a contact time of 120 s each. The concentration for each protein was chosen to achieve the maximal possible saturation, which was approximately 70% occupancy of mesothelin molecules for CA-125 and 100% for the anti-mesothelin Fab. Maximal saturation was calculated as follows, the ratio R_max_ was defined as R_max_ experimental/R_max_ theoretical, where 100% theoretical response maximum is calculated from the capture level of the first interaction partner (as the response units are directly proportional to molecular weight) assuming two binding sites for each dimeric MSLN Fc fusion protein. Saturation was demonstrated by a 2nd control injection of higher concentrated protein that did not further raise response level. Capture antibody was regenerated with 3 M MgCl_2_ as recommended by the vendor. Experiments were done at 25 °C to minimize dissociation.

### MSLN FACS analysis

Cell surface MSLN levels of the NCI-H358 clones were measured by flow cytometry. 5 × 10^5^ cells were incubated with 10 µg/ml of the humanized SS1P IgG. After washing, the primary antibody was detected with an anti-human Alexa Fluor 647 antibody (Thermo Fisher Scientific, Darmstadt, Germany). The labelled cells were washed, fixed and fluorescence intensities quantified by FACs.

### Heterotypic cell adhesion assay

An assay protocol described by Rump *et al*., 2004 was adapted. On the first day of the assay, 1 × 10^6^ OVCAR3 cells were seeded per well of a 12-well dish. After washing with PBS, 1 × 10^7^ NCI-H358 cells were labelled with 2.5 µM CFSE for 10 min at 37 °C, then the reaction was stopped with FCS, and the cells were cultured overnight. On the following day the NCI-H358 cells were counted and 1 × 10^6^ cells were pre-incubated with 20 µg of anti-MSLN Fab or isotype control for 10 min at 25 °C. The NCI-H358 cells were then added to the OVCAR3 cells in the 12-well dish and the plates were centrifuged for 3 min at 150x g. Afterwards the wells were washed 5 times with PBS under agitation. The cells were incubated with enzyme free cell dissociation buffer (EFCDB: PBS with 5 mM EDTA, 1 mM sodium pyruvate, 10 mM HEPES buffer) until they could be gently washed off the plate, collected, and analyzed by flow cytometry.

### CellTiter-Glo assay

1 × 10^4^ NCI-H226 cells, in 50 µl were seeded in wells of a white walled 96-well plate with or without CA-125 and incubated overnight. PBS was added to the outside rows of the plate as well as the space between the wells to reduce evaporation. On the following day, 1:3 serial dilutions of RG7787 were made starting at 3 µg/ml. Serial dilutions were also made of a control Fab-PE that exerts potent cytotoxic activity for the target cells via a surface target that, unlike mesothelin, does not interact with CA-125. 50 µl of each dilution was added to the wells. After 72 h incubation cell viability was determined using CellTiter-Glo^®^ reagent and plotted as percent inhibition versus untreated controls.

### RG7787 sensitivity screening of ovarian cancer cell lines

Freshly thawed and expanded cell lines that had reached their optimal doubling time were seeded at a density of 5 × 10^2^ cells per well in black-walled 384-well tissue culture plates and incubated at 37 °C for twenty‐four hours before treatment. Potency of RG7787 on the different cell lines was determined in quadruplicate using ten point titration curves with a 1:3 serial dilution starting at 50 nM. Cellular ATP content was determined after 72 hours using ATPlite (Perkin Elmer; Rodgau, Germany). As reference points for the values (T) determined after 72 hours incubation with RG7787 the ATP levels (V_0_) of a set of untreated assay plates were also measured at time point zero. Percent growth inhibition (GI) as a measure of cell growth and cell death was calculated with the following formulas:

$$\begin{array}{c}{\rm{If}}\,{\rm{T}} < {{\rm{V}}}_{0}: \% {\rm{GI}}=100\times (1\mbox{--}({\rm{T}}\mbox{--}{{\rm{V}}}_{0})/{{\rm{V}}}_{0})\\ {\rm{If}}\,{\rm{T}}\ge {{\rm{V}}}_{0}: \% {\rm{GI}}=100\times (1\mbox{--}({\rm{T}}\mbox{--}{{\rm{V}}}_{0})/({\rm{V}}\mbox{--}{{\rm{V}}}_{0}))\end{array}$$

T is the signal measured at 72 hours. V is the signal of the vehicle-treated control wells measured at 72 hours. V0 is the signal of untreated control wells measured at time point zero. A GI reading of 0% represents no growth inhibition (i.e. T = V), while 100% GI indicates complete growth inhibition (i.e. T = V_0_). A GI of 200% reflects complete death of all cells in the culture well (full cytotoxicity) and results from T ≪ V0 so that T − V0 reaches values near zero.

### Immunohistochemistry analysis of OvCa6668 tumors

Formalin fixed paraffin embedded tissue blocks were obtained from Experimental Pharmacology & Oncology (EPO, Berlin, Germany). Tissue slices of 2.5 µm thickness were transferred onto glass slides. A rabbit monoclonal antibody against human mesothelin (clone SP74, Spring Bioscience, Pleasanton, USA) was applied at a concentration of 1 µg/ml. Automated staining was performed on a Ventana Benchmark XT. Staining for CA-125 was performed applying a monoclonal rabbit anti-CA-125 antibody (clone EPR1020, Ab110640; Cambridge, UK) at a concentration of 0.3 µg/ml on a Ventana Discovery XT.

### Efficacy studies in an ovarian patient-derived xenograft (PDX) model

The *in vivo* efficacy studies with an ovarian cancer PDX model (OvCa6668) were performed at EPO (Berlin, Germany). All animal experiments were performed in accordance with the general principles governing the use of animals in experiments of the European Communities and the German legislation. The study was done in accordance with the United Kingdom Coordinating Committee on Cancer Research regulations for the Welfare of Animals^38^ and of the German Animal Protection Law and were approved by the local responsible authorities, Berlin, Germany (Approval No. A 0452/08, LaGeSo Berlin, Germany). OvCa6668 subcutaneous tumors were inoculated in NMRI nu/nu female mice. On day 15, when tumors had reached sizes of 80–250 mm^3^, animals were randomized into treatment groups (n = 10) with comparable ranges of tumor sizes. Treatment began on day 16 and animals were dosed with either 6 mg/kg i.v. paclitaxel daily for 5 days (q1dx5), 2 mg/kg i.v. RG7787 dosed every other day for 2 weeks plus a final dose in week 3 (q2dx3 2 days off, q2dx3 2 days off, a plus 7th treatment), 2 mg/kg i.p. cisplatin dosed once weekly for 2 weeks (q7dx2) or the combination or RG7787 with each chemotherapy. The combinations were performed with the same dose schedules as described above. Tumor volumes were determined by standard caliper measurements. All monotherapies were well tolerated (body weight loss 5–10%). To assess synergistic effects of combination treatment we calculated the hypothetical tumor growth curve that would result from a purely additive effect based on the “Bliss independence” model^39,40^.

### Statistical Methods

For the *in vitro* potency assays the concentration curves were fitted using the sigmoidal dose-response model in XLfit (Version 5.5.0.5). The IC50 values and the corresponding 95% confidence intervals were extracted from the fitted models. No overlap between the CI of two curves indicates a statistically significant difference between the two IC50 values.

For the xenograft experiments the data were processed in the statistics software SAS-JMP version 8.0.2.2 (SAS Inc., Cary, NC, 2007). Since the data regarding primary tumor growth showed asymmetrical distribution, they were analyzed using non-parametric statistical methods^41^. Pairwise comparisons were conducted using the Steel-Dwass method which corrects for multiple testing.

The single agent inhibition values were used to calculate the expected effect under the assumption of additivity. The additive effect was calculated using the Bliss model of additivity: additive effect = mean of vehicle*(1-((1-SingleAgent1) + (1-SingleAgent2)-(1-SingleAgent1)*(1-SingleAgent2))). Details of Bliss additivity model can be found in previous publications^40,42^. We used the Bliss model in this study because it assumes independent mechanisms of action of the two drugs. Based on this model, a synergistic drug combination is a combination that produces an effect that is stronger than the anticipated additive effect.

### Data Availability

For the current study datasets were analyzed from the following publically available data sources: (i) the Cancer Genome Atlas (NIH) and (ii) the Cancer Cell Line Encyclopedia (Broad Institute). For DPH1 gene analysis data were also obtained from a Roche internal cell bank data base.

## Electronic supplementary material

Supplementary Information
