# Supplementary material for: A re-engineered immunotoxin shows promising preclinical activity in ovarian cancer
Source: Sci Rep. 2017 Dec 22;7:18086. doi: 10.1038/s41598-017-17329-7 (PMC5741731; doi:10.1038/s41598-017-17329-7)
Supplement: Supplementary file 1 — Supplementary Information [file 41598_2017_17329_MOESM1_ESM.pdf]

A re-engineered immunotoxin shows promising preclinical activity in ovarian cancer

Gwendlyn Kollmorgen, Klara Palme, Annette Seidl, Stefan Scheiblich, Fabian Birzele, Sabine Wilson, Christian Clemens, Edgar Voss, Martin Kaufmann, Klaus Hirzel, Natascha Rieder, Ben-Fillippo Krippendorf, Frank Herting, Gerhard Niederfellner

Figure S1A and S1B Prevalence of mesothelin and CA125 in different cancer types. The Human Cancer Genome Atlas was mined for the expression levels of MSLN (Fig. S1a) and CA125 (Fig. S1b) in different human tumor types compared to respective normal tissues. For each indication, target expression is shown as box and whisker plot for normal tissue on the left and for the corresponding tumor tissue on the right. Numbers in brackets below the graphs indicate how many samples were analyzed for normal (N) and tumor (T) tissue. Indications with a statistically significant difference in the median target expression level between normal and tumor tissue (either increased or decreased) are marked by an asterisk and the difference in log2 values is indicated as “Effect (T-N)”.

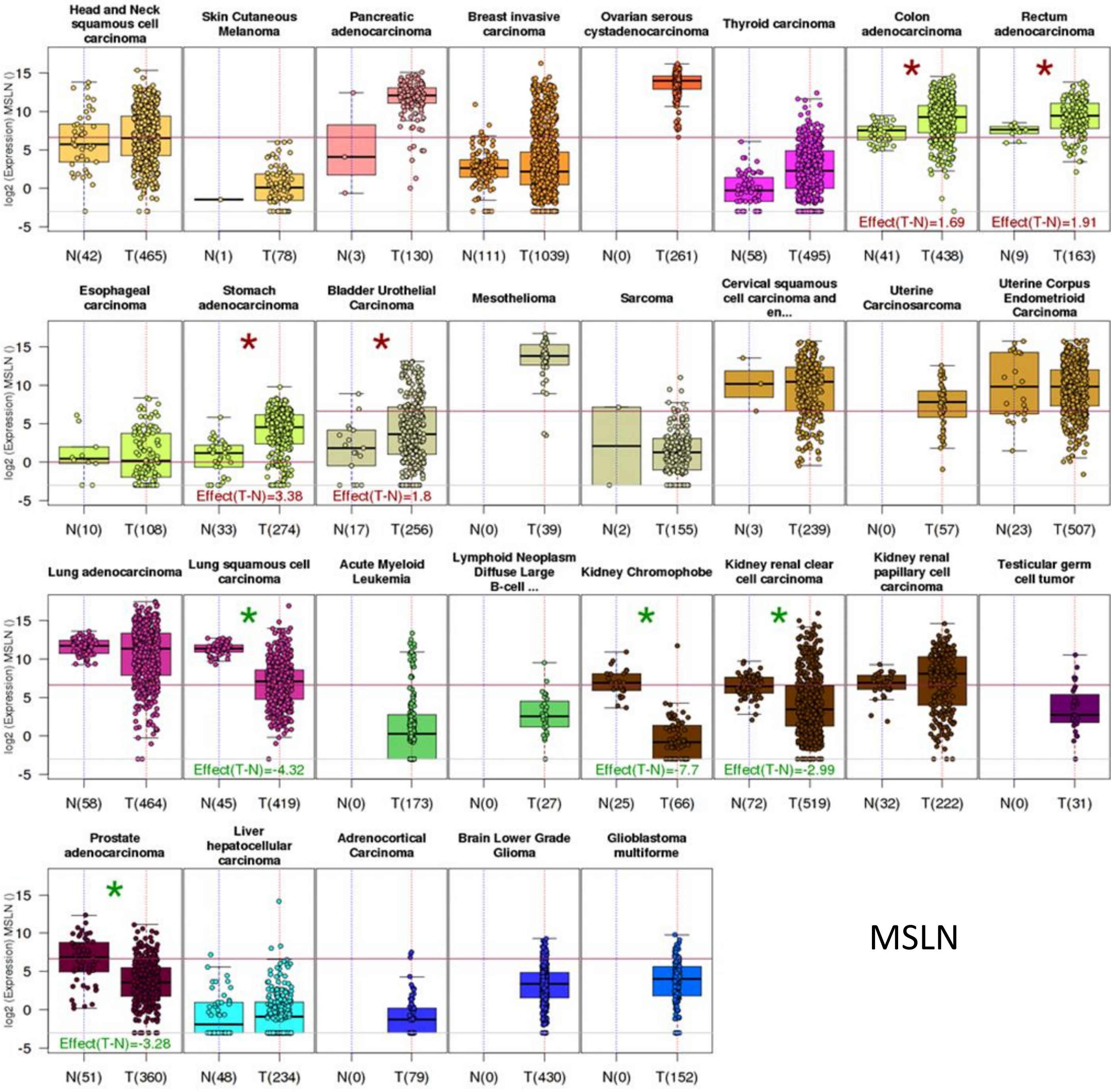
Head and Neck squamous cell carcinoma
Skin Cutaneous Melanoma
Pancreatic adenocarcinoma
Breast invasive carcinoma
Ovarian serous cystadenocarcinoma
Thyroid carcinoma
Colon adenocarcinoma
Rectum adenocarcinoma
log2 (Expression) MSLN ()
Effect(T-N)=1.69
Effect(T-N)=1.91
N(42) T(465)
N(1) T(78)
N(3) T(130)
N(111) T(1039)
N(0) T(261)
N(58) T(495)
N(41) T(438)
N(9) T(163)
Esophageal carcinoma
Stomach adenocarcinoma
Bladder Urothelial Carcinoma
Mesothelioma
Sarcoma
Cervical squamous cell carcinoma and en...
Uterine Carcinosarcoma
Uterine Corpus Endometrioid Carcinoma
Effect(T-N)=3.38
Effect(T-N)=1.8
N(10) T(108)
N(33) T(274)
N(17) T(256)
N(0) T(39)
N(2) T(155)
N(3) T(239)
N(0) T(57)
N(23) T(507)
Lung adenocarcinoma
Lung squamous cell carcinoma
Acute Myeloid Leukemia
Lymphoid Neoplasm Diffuse Large B-cell ...
Kidney Chromophobe
Kidney renal clear cell carcinoma
Kidney renal papillary cell carcinoma
Testicular germ cell tumor
Effect(T-N)=-4.32
Effect(T-N)=-7.7
Effect(T-N)=-2.99
N(58) T(464)
N(45) T(419)
N(0) T(173)
N(0) T(27)
N(25) T(66)
N(72) T(519)
N(32) T(222)
N(0) T(31)
Prostate adenocarcinoma
Liver hepatocellular carcinoma
Adrenocortical Carcinoma
Brain Lower Grade Glioma
Glioblastoma multiforme
Effect(T-N)=-3.28
N(51) T(360)
N(48) T(234)
N(0) T(79)
N(0) T(430)
N(0) T(152)
15
10
5
0
-5

MSLN

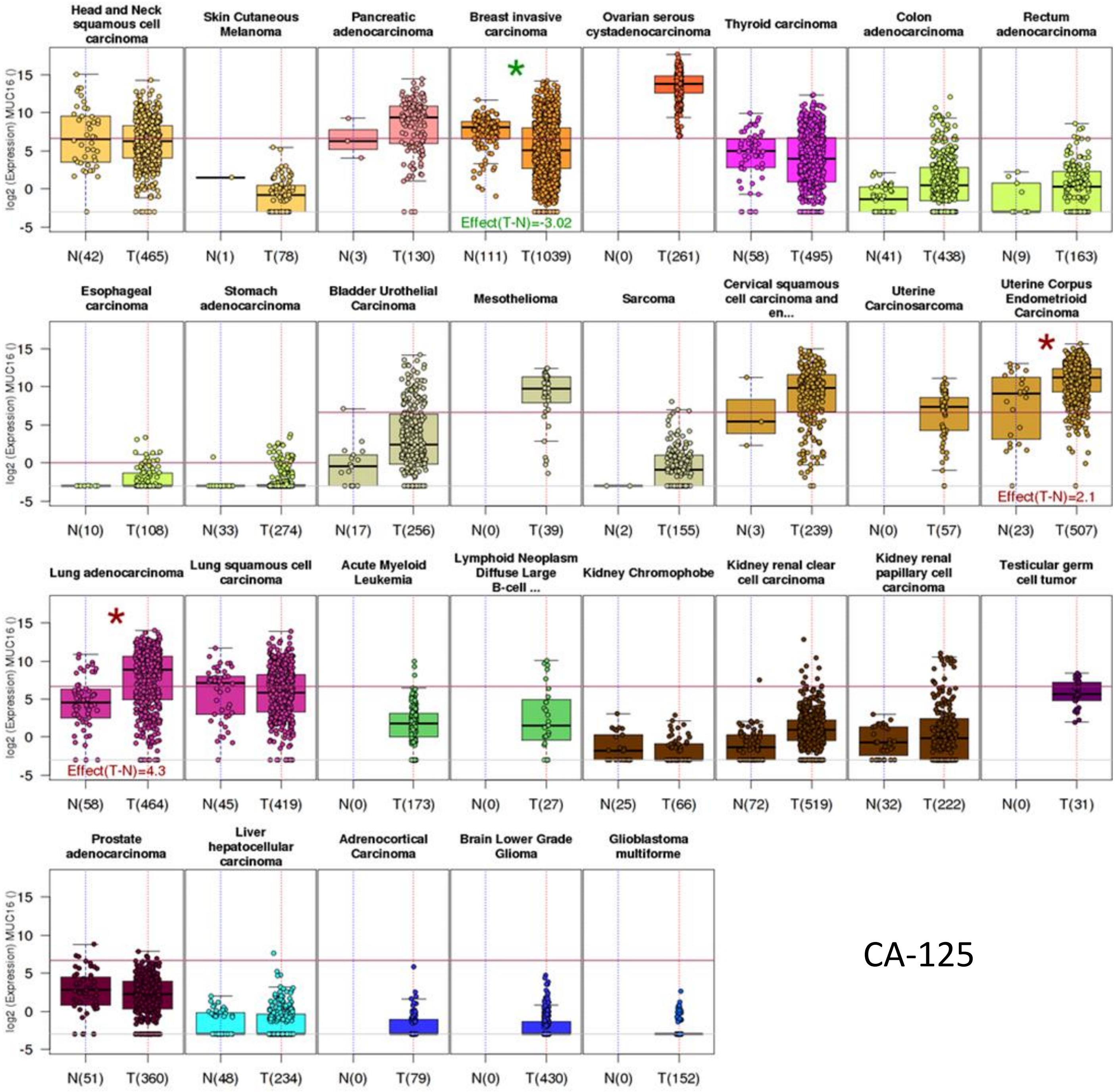
Head and Neck squamous cell carcinoma
Skin Cutaneous Melanoma
Pancreatic adenocarcinoma
Breast invasive carcinoma
Ovarian serous cystadenocarcinoma
Thyroid carcinoma
Colon adenocarcinoma
Rectum adenocarcinoma
log2 (Expression) MUC16 ()
Effect(T-N)=-3.02
N(42) T(465) N(1) T(78) N(3) T(130) N(111) T(1039) N(0) T(261) N(58) T(495) N(41) T(438) N(9) T(163)
Esophageal carcinoma
Stomach adenocarcinoma
Bladder Urothelial Carcinoma
Mesothelioma
Sarcoma
Cervical squamous cell carcinoma and en...
Uterine Carcinosarcoma
Uterine Corpus Endometrioid Carcinoma
Effect(T-N)=2.1
N(10) T(108) N(33) T(274) N(17) T(256) N(0) T(39) N(2) T(155) N(3) T(239) N(0) T(57) N(23) T(507)
Lung adenocarcinoma
Lung squamous cell carcinoma
Acute Myeloid Leukemia
Lymphoid Neoplasm Diffuse Large B-cell ...
Kidney Chromophobe
Kidney renal clear cell carcinoma
Kidney renal papillary cell carcinoma
Testicular germ cell tumor
Effect(T-N)=4.3
N(58) T(464) N(45) T(419) N(0) T(173) N(0) T(27) N(25) T(66) N(72) T(519) N(32) T(222) N(0) T(31)
Prostate adenocarcinoma
Liver hepatocellular carcinoma
Adrenocortical Carcinoma
Brain Lower Grade Glioma
Glioblastoma multiforme
N(51) T(360) N(48) T(234) N(0) T(79) N(0) T(430) N(0) T(152)

CA-125
